# Obesity Treatment Application Implications of Temporally Sequenced Paths of Theory-Driven Psychological Changes Toward Improvements in Physical Activity and Dietary Behaviors in Women

**DOI:** 10.3390/nu18030391

**Published:** 2026-01-24

**Authors:** James J. Annesi

**Affiliations:** Kinesiology Department, School of Health Sciences and Human Services, California State University, Monterey Bay, 100 Campus Center, Seaside, CA 93955, USA; jamesannesi@gmail.com; Tel.: +1-404-579-1809

**Keywords:** self-regulation, self-efficacy, mood, physical activity, obesity, behavioral, treatment, weight loss

## Abstract

**Background/Objectives:** Obesity has a high prevalence and is associated with many health risks. Minimal effects from behavioral obesity treatments might be linked to their atheoretical dependence on simply educating participants on healthy eating and increased physical activity/exercise, rather than evolving behavior-change methods through theory-based research. The use of pharmacologic interventions has recently overtaken bariatric surgery in medically based efforts to obtain greater weight losses than through behavioral means. **Methods:** The present longitudinal observational study aimed to extend earlier treatment-associated findings concerned with the order of emphasizing 3-, 6-, and 9-month changes in the theory-driven psychosocial constructs of self-regulation, self-efficacy, and mood associated with 12-month improvements in weight-loss behaviors. The “parent study” of women with obesity (*N* = 106) found significant paths through changes in self-regulation → mood → self-efficacy and mood → self-regulation → self-efficacy. In the present extension of that investigation, only those participants who did not already complete recommended physical activity amounts and consume recommended portions of fruits/vegetables at baseline were included (*N* = 73). **Results:** Only paths from changes in mood → self-regulation → self-efficacy were significantly associated with 12-month improvements in both physical activity and dietary behaviors. A mean weight change of −5.2 kg, or −5.5% from baseline weight, was found. Baseline scores in emotional eating and anxiety significantly moderated the mood–self-regulation change relationships within the significant paths. **Conclusions:** Findings suggested that future treatment contents focus on early physical activity-associated improvement in mood because of its association with increased usage of treatment-developed self-regulatory skills. Those skills should then be leveraged because of their association with feelings of ability (i.e., self-efficacy) to overcome lifestyle barriers to weight-loss behavior changes. Further improvements in behavioral obesity treatments should be reconsidered as either stand-alone modalities or, after appropriate testing, as an adjunct to medical means.

## 1. Introduction

Obesity (body mass index [BMI] ≥ 30 kg/m^2^) is a medical condition associated with a plethora of health risks [1]. Although continuing to increase worldwide for many decades, obesity is especially prevalent in countries with advanced economies [2]. In the United States (U.S.), for example, obesity is present in 46% of women of at least 25 years and expected to rise to 59% by 2050 [3]. Although controlling one’s eating behaviors and increasing physical activity (and its more structured form termed “exercise”; PA) will reliably reduce excess weight [4], those behaviors have been *extraordinarily* difficult to maintain beyond the short term—especially when the typical methods of education on healthy eating (possibly through prescribed “diets”) and exercise (possibly through specific workout regimens) are relied upon [5,6]. Effects of ~5% (and up to 8%) loss from baseline weight were suggested when behavioral treatment guidelines focus upon such lifestyle change [7]. Inclusion of low-compliers has, however, been variable within the associated research which calls into question those rates of lost weight. Even in the 9% most compliant of its overall 500,000 participants, a mean loss of <3% was observed using the nationally (U.S.) administered MOVE! Behavioral Weight Management Program [8]. That result is consistent with administrations of the well established LEARN Program for Weight Management [9], which recorded losses of 1.9% [10] and 2.4% [11] from baseline weight. Beyond around 6 months, where a plateau in lost weight is reliably predicted, a progression to near complete regain is common [5,12].

Consequently, both weight-loss surgeries and medication usage have increased [13,14]. Reasons for the recent upsurge in use of glucagon-like peptide-1 (GLP-1) receptor agonists—a class of pharmacologics that includes semaglutide (e.g., Wegovy, Ozempic) and tirzepatide (e.g., Zepbound, Mounjaro)—over surgeries and other medication types have been suggested [14,15]. As with participation in weight-loss treatments generally, women [16], especially those of the higher socioeconomic strata for the medical means [17,18], are the most frequent recipients. Although an aggregate of randomly controlled trials suggested 12–18% weight loss through GLP-1 usage [19,20], research using “real-world” data (where treatment compliance is more variable, and non-adherent data are included) indicated a mean loss of 4.5% over a year [21]. Although some suggest that surgeries and medications are transformative in the treatment of obesity [22], and work on behavioral (non-medical) approaches should be terminated due to their continued unsatisfactory outcomes [11], others posited that improved research may still enable meaningful progress as long as traditional and atheoretically based methods are supplanted [23,24,25]. If enhanced, behavioral interventions might also be more applicable within largescale settings—across socioeconomic groupings—because of their minimal expense, need for medical staffing, and risk to participants [26]. There is a lack of research on the real-world effects of GLP-1 paired with behavioral methods, with the certainty of the two identified studies on that topic being classified as “low to very low” [27]. It should be noted; however, that both the explanatory mechanisms and clinical objectives are distinct across the different weight-loss intervention types.

Some research suggests that better addressing theory-based psychosocial constructs such as those associated with individuals’ internal regulatory processes will be useful for improving behavioral obesity treatments [28]. Given the indications that PA, along with dietary change, is crucial for sustained weight loss, a predictive model was proposed suggesting that PA influences weight loss through associated psychosocial correlates of improved eating [29]. The variables proposed as salient within that conceptual model were: (a) physical self-concept, (b) mood, (c) coping/self-regulation, (d) body satisfaction/body image, and (e) self-efficacy (i.e., feelings of ability) [29]. Influenced by that paradigm, a systematic research program of 25 years indicated that there were *three* key psychosocial variables [30]. Those were self-regulation, overall mood, and self-efficacy, which have considerable overlap with the Baker and Brownell model [29]. Research indicated that when changes in those variables were entered into regression models, each contributed a unique portion of the variance in both PA and dietary behaviors over the short-term (e.g., 3 months), longer-term (e.g., 6–9 months), and long term (e.g., 12–24 months) [30].

Social cognitive theory [31,32] and self-efficacy theory [33]—which posit individuals’ potential to overcome their environmental challenges and barriers to health behavior changes through self-management and associated feelings of ability—were the basis of formal testing of both that three-factor model and effects of concomitant treatments on excess body weight [30]. Additional theories served as extensions of social cognitive theory and self-efficacy theory and helped clarify interrelations among the included factors. For example, the mood–behavior model [34] suggested a positive effect of the expected exercise-associated mood improvements [35] on self-regulating eating and sustaining PA [36]. Also, self-regulation theory [37] guided treatment methods through participants’ regular rehearsal of specific self-regulatory skills (that have been suggested as consistent with social cognitive theory [38]). In accordance with coaction theory [39], increases in PA were initially pursued so that its associated psychological changes could subsequently be transferred (generalized) to dietary changes [30]. The focus on PA to support changes in psychosocial correlates of eating-behavior change, rather than its limited energy expenditure potential in adults with obesity where adherence is a concern if exercise regimens are excessive [40,41], was a novel treatment aspect.

As the research program progresses, and further precision is sought to improve its shaping of future intervention contents, optimal temporal patterns for treatment emphases are considered. Recently, paths incorporating the full matrix of 3-month → 6-month → 9-month changes in merged (PA- and diet-related) measures of mood, self-regulation, and self-efficacy—leading to 12-month changes in PA and overall dietary change—were tested [42]. Those analyses were of women with obesity seeking weight loss within a community setting. That research [42] is referred to within this report as the “parent study.” Findings indicated that, of the many possible combinations, only changes from self-regulation → mood → self-efficacy and mood → self-regulation → self-efficacy significantly predicted both PA and dietary change. Thus, each significant path initiated from an interaction of changes in self-regulation and mood. Within National Institutes of Health/National Cancer Institute-certified treatment processes associated with that behavioral research (devoid of surgical and/or pharmacologic methods [10]), weight loss in women was 5.8% at the 12-month point. That intention-to-treat finding demonstrated a notable 18% more loss than the real-world evaluation of the reviewed GLP-1 pharmacological approach in women [21,43].

The parent study suggested *when* it was most advantageous to emphasize which psychosocial change within its sample type, which was a valuable contribution. That research, however, included participants who, at baseline, were already completing the recommended daily intake of five portions of fruits/vegetables (a proxy for the health of the overall diet [44,45]) and the recommended amount of weekly PA of 150 min [46]. Thus, the treatment’s primary behavioral goals were less relevant to that study’s participants and could have compromised the applicability of the overall results. Therefore, to extend those findings [42], the present research included only participants who were not already completing the aforementioned behavioral targets at treatment start. Also, based on previous research [47], relationships between changes in overall mood and self-regulation could have been affected by participant-specific variables such as emotional eating, anxiety, and depression at treatment initiation. Therefore, because of possible intervention implications, those scores were additionally tested here as moderators of the mood–self-regulation change relationship.

Aims of this study were to extend an ongoing progression of theory-based research [30] and further refine obesity treatment foci on mood, self-regulation, and self-efficacy from a temporal perspective. Hopefully, findings will facilitate an increase in both the reliability and amount of weight loss capable within a field setting using behavioral means. Hypotheses are as follows:

1. Psychosocial scores will significantly improve over 3 and 6 months; however, it was unclear whether score changes occurring beyond 6 months would negate the positive effects.
2. Paths from changes in overall mood → self-regulation → self-efficacy → PA and dietary behaviors will be significant (in separate models). Paths from changes in self-regulation → mood → self-efficacy → PA and the diet will also be significant.
3. Within the path models demonstrating significance, relationships between changes in mood and self-regulation will be significantly moderated by participants’ baseline score on emotional eating, anxiety, and depression.
4. There will be a significant inverse association between changes in participants’ PA and overall negative mood and, over the 12-month duration of the study, between improvements in their PA and dietary behaviors, and weight.

## 2. Materials and Methods

### 2.1. Participants

Participants were part of an ongoing field-based course of research conducted within U.S. community settings. The associated investigations aimed to improve behavioral weight-management methods in those with excess weight through a systematic progression of theory-driven studies. Women of at least 21 years of age with obesity volunteered for the present research by responding to local newspaper advertisements and social media. There was no cost or financial compensation for participating. Inclusion criteria were: (a) adequate physical condition for safe and full participation; (b) no current/soon-planned pregnancy; and (c) no weight-loss plan/program participation (including self-administered), nor start/change in a psychotropic medication, within the previous 12 months. The initially self-reported weight/height ratio (i.e., BMI) indicative of obesity was cross-checked by study staff measurements before study start. Also, the present sample was limited to those women of the parent study who neither consumed the minimum recommended daily portions of fruits/vegetables (i.e., five) nor completed the minimum recommended amount of weekly PA (i.e., 150 min) at baseline (as measured by the corresponding self-report instruments in the below Section 2.2). Based on those additional criteria, the parent study sample of 106 participants [42] was reduced to 73, which was adequate for the planned primary regression analyses (see the Data Analyses Section 2.4). The age range was 21–59 years (*M* = 46.4 years, *SD* = 10.1); the BMI range was 30.0–40.7 kg/m^2^ (*M* = 34.2 kg/m^2^, *SD* = 3.2); the racial/ethnic make-up was 64% White, 25% Black, and 11% Hispanic; and the educational levels were 81% bachelor’s degree or greater and 19% high school or college. All but two participants self-reported within the middle yearly family income range of USD 50,000–USD 150,000. Ethical requirements of the Declaration of Helsinki and the American Psychological Association were upheld. A university institutional review board approved the study protocol and the informed consent process requiring a signature from each participant.

### 2.2. Measures

An aggregated measure of PA-related and controlled eating-related self-efficacy (Self-eff-Ag) was scored incorporating (a) the 5-item Exercise Self-Efficacy Scale (e.g., “I am tired”) [48] and (b) the 20-item Weight Efficacy Life-Style Questionnaire (e.g., “I can resist eating even when high-calorie foods are available”) [49]. After minor adjustments for scoring symmetry across scales, possible responses ranged from 1 (*not confident*) to 10 (*very confident*), with mean item scores recorded after adjusting for the difference in the number items in each scale. Reported internal consistencies ranged from Cronbach’s α = 0.76–0.82 [48], and Cronbach’s α = 0.74–0.78 [49], respectively. For the present scale and sample, Cronbach’s α = 0.76.

An aggregated measure of PA-related and controlled eating-related self-regulation (i.e., use of specific self-management skills; Self-reg-Ag) integrated the Exercise-Related Self-Regulation Scale (e.g., “I make formal agreements with myself to be physically active”) and the Eating-Related Self-Regulation Scale (e.g., “I keep a record of my eating”), both having 10 items [50]. Possible responses ranged from 1 (*never*) to 4 (*often*), with mean item scores recorded. Reported internal consistencies were Cronbach’s α = 0.79 and 0.81, respectively and test–retest reliabilities over 2 weeks were 0.78 and 0.74, respectively [50]. For the present scale and sample, Cronbach’s α = 0.75.

The 30-item Profile of Mood States-B [51] measured overall negative mood (OverallNegMood) through an aggregation of its six subscales of 5 items of one to three words each. They were anxiety (“tense”), depression (“gloomy”), fatigue (“sluggish”), confusion (“bewildered”), anger (“angry”), and vigor (“active”). Possible responses ranged from 0 (*not at all*) to 4 (*extremely*). Reported internal consistencies for women ranged from Cronbach’s α = 0.84–0.95 (anxiety and depression subscales were Cronbach’s α = 0.90 and 0.95, respectively). Test–retest reliabilities over a median of 3 weeks were 0.65–0.74 [51]. For the present sample, Cronbach’s α = 0.82–0.94.

Emotional eating was measured through 15 items of the Emotional Eating Scale [52]. They addressed dimensions of anxiety (“nervous”), depression (“sad”), and anger/frustration (“irritated”) related to prompts to eating. Possible responses ranged from 0 (*no desire to eat*) to 4 (*an overwhelming urge to eat*), which were summed. Reported internal consistencies ranged from Cronbach’s α = 0.72–0.79, and the test–retest reliability over 3 weeks was 0.79 [52]. For the present sample, Cronbach’s α = 0.74.

PA was measured using the Godin-Shephard Leisure-Time Physical Activity Questionnaire [53]. The number of sessions of “mild intensity” (e.g., normal-paced walking), “moderate intensity” (e.g., fast-paced walking), and “strenuous intensity” (e.g., running) PA with a duration of at least 15 min during the previous 7 days were recalled. Scores of 3, 5, or 9 metabolic equivalents (METs) were allocated, respectively. A MET is a measure of energy expenditure beyond the resting state. After multiplying the number of sessions by its corresponding MET value, they were summed. A score of 25, for example, corresponds to 5 sessions/week at a moderate intensity level. Godin-Shephard Leisure-Time Physical Activity Questionnaire scores exhibited considerable validity through their relationships with accelerometry, body composition, and VO_2_ max cardiovascular test results, with 2-week test–retest reliability at 0.74 [54,55,56,57,58]. The recall instrument has frequently been assimilated into medical research [59].

Overall dietary behavior (Diet-overall) was assessed using a previously applied method of aggregating recalled food intake over a 7-day period where its scoring weights the positive effects of combined fruit and vegetable intake (×2) minus the detrimental effects of sweets (×1) [42,44,60,61]. While adjusting for factors such as consumption of large or small amounts, added fats/sugars in preparation, and combination foods (e.g., salads), portion sizes were based on U.S. governmental sources [62,63]. Examples were a moderate-size (118 mL) apple, moderate amount (118 mL) of green beans, small (59 mL) cookie, and a cup (237 mL) of full-sugar soda. The 3-week test–retest reliabilities in women were reported at 0.77–0.83, and concurrent validity was demonstrated through correspondences with lengthier and well validated food recalls [64,65].

Weight was measured to the nearest 0.10 kg using a medical grade digital scale (Health-O-Meter 80KL; McCook, IL, USA) that was re-calibrated the day of each measurement. Participants removed their shoes and heavy outer clothing prior to measurement. To enable calculation of the BMI inclusion criterion, height was measured to the nearest 0.10 cm using a stadiometer (Health-O-Meter Portrod; McCook, IL, USA). All measurements were conducted by non-instructional study staff.

### 2.3. Procedure

Treatment instructors were all existing staff members of the community wellness centers participating in the current research, with each indicating a desire to be involved in the present project. They were trained by study staff in the year-long behavioral treatment protocol that was based on obesity programing certified by the National Institutes of Health/National Cancer Institute, requiring ~25 h from each participant. The self-regulation skill-based protocol was guided, overall, by social cognitive theory [31] and self-efficacy theory [33]. However, other paradigms supported specific procedural aspects of the treatment such as the (a) increase and improvement of self-regulatory skills usage through practice (self-regulation theory [37,66], (b) interaction of PA-associated mood change with self-regulation (mood–behavior model [34]), and (c) generalization of psychological mechanisms of PA change to those of an improved diet (coaction theory [39]). Each of those theoretical models emphasized how weight-loss behaviors could be positively affected by treatment attention placed on environmental, psychosocial, and behavioral factors. There was a combination of 45 to 50 min individual and small-group instruction sessions convening every 2 weeks after more closely spaced weekly meetings initially.

The suggested self-regulatory skills targeted for development included goal setting/proximal progress tracking, cognitive restructuring, relapse prevention, stimulus control, and attention control (e.g., dissociation from discomfort) [38]. Increase in self-efficacy was sought though an emphasis of self-regulatory skill usage helping to successfully manage formidable behavioral challenges. Generalization of self-management processes across PA and dietary behaviors was also pursued throughout. While the suggested ≥150 min/week of at least moderate PA for health benefits [46] was stated to participants, simply increasing PA using their preferred modalities was the imposed goal. Goals for dietary behaviors centered around increasing fruit/vegetable intake, minimizing sweets, and maintaining 1200–1500 kilocalories/day (which were presented as directives, but not monitored). Because of research indicating its benefits [16], weekly self-weighing was also suggested to participants by instructors. Participants were referred to the U.S. government website, http://myplate.gov/, for additional nutrition information when or if desired.

In-person fidelity checks were conducted by non-instructional study staff using a structured form. Strong protocol compliance was detected, with the minor issues addressed by direct staff-instructor interaction. The same non-instructional study staff members administered measurement instruments to participants at the designated intervals in a private area. Collected data were kept anonymous and confidential.

### 2.4. Data Analyses

There was no systematic bias [67] found in presence/absence of the 14% of missing data, each of those datum were beyond baseline. That was evidenced by no significant difference (*p*s > 0.30) between participants with vs. without any of the present psychological or behavioral scores missing within the analyses. That missing-at-random condition fulfilled criteria for incorporation of the expectation-maximization algorithm for imputation, facilitating an intention-to-treat format [68,69]. For the primary regression analyses, a sample size of ≥70 participants was required to detect the effect of Cohen’s *f*^2^ = 0.20 (derived from related research [30]) at the statistical power of 0.80, α < 0.05 [70]. Variance inflation factor values of <2.00 indicated no adverse issues with multicollinearity, and there were no observed floor or ceiling effects. This sample’s results were from a post hoc subselection of the parent study.

Dependent *t* tests were first used to assess significance of within-group gains (changes; Δ) in the measures of Self-reg-Ag, OverallNegMood, and Self-eff-Ag from baseline–Months 3, 6, and 9, and changes in PA and Diet-overall from baseline–Month 12. Next, based on the aims of this research and the previously identified significance of two of the six tested pathways of psychosocial changes (i.e., Δbaseline–Month 3 → Δbaseline–Month 6 → Δbaseline–Month 9) toward 12-month changes in both PA and Diet-overall identified within the parent study [42], the paths evaluated here were changes in Self-reg-Ag → OverallNegMood → Self-eff-Ag → PA, Self-reg-Ag → OverallNegMood → Self-eff-Ag → Diet-overall, OverallNegMood → Self-reg-Ag → Self-eff-Ag → PA, and OverallNegMood → Self-reg-Ag → Self-eff-Ag → Diet-overall. Thus, unlike in the previous study, the current investigation was separately concerned with paths toward changes in PA and Diet-overall.

To assist findings in shaping intervention contents with improved precision, within any path defined as significant within the present analyses, baseline emotional eating, anxiety, and depression scores were next separately entered as a moderator of relations between ΔOverallNegMood and ΔSelf-reg-Ag. Those findings might have treatment ramifications based on those personal characteristics common in women with obesity [71]. As suggested, within the corresponding analyses, both the moderation terms of the ΔOverallNegMood–ΔSelf-reg-Ag relationship, and the index of moderated mediation (IMM, which accounts for moderation as part of the overall model [72]), were calculated.

Finally, to evaluate associations of PA with mood within the present context, sensitivity analyses assessed linear relationships between both baseline PA and 3-month change in PA, and change in OverallNegMood over 3 months. Also, to evaluate associations of the target behaviors on excess weight, 12-month changes in PA and Diet-overall were simultaneously entered as independent variables within a multiple regression equation for their relationship with weight change over 12 months. Statistical assessments were implemented using SPSS Statistics version 28.0.1.0 (IBM Corp., Armonk, NY, USA) along with the PROCESS version 4.2 macroinstruction Models 6 and 83, incorporating 10,000 percentile-based bootstrapped re-samples of the data [72]. Statistical significance was set at α < 0.05 (two-tailed). Where bootstrapping was incorporated, a 95% confidence interval (95% CI) was used to determine statistical significance. Due to bases in theory, and congruent with suggestions for similar analyses [73,74], there was no adjustment of significance level for multiple tests.

## 3. Results

### 3.1. Score Changes in Study Data

Descriptive data and change scores of the included variables at their assessed intervals are given in Table 1. Significant improvements (*p*s < 0.001) associated with the treatment were found in the paired analyses, with large effect sizes ranging from *d* = 0.86–1.60. The only exceptions were declines in Self-eff-Ag and Self-reg-Ag over 9 months where scores associated with their Month 6–9 decreases negated the significant progressive gains made earlier.

### 3.2. Significance of Proposed Paths Toward Behavioral Changes

**Figure 1:**
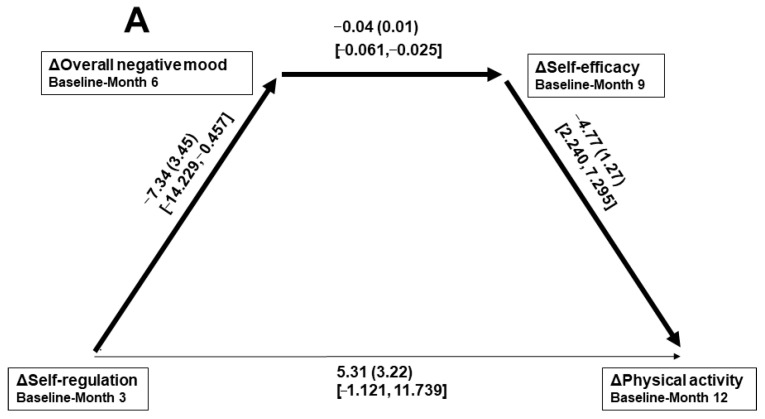

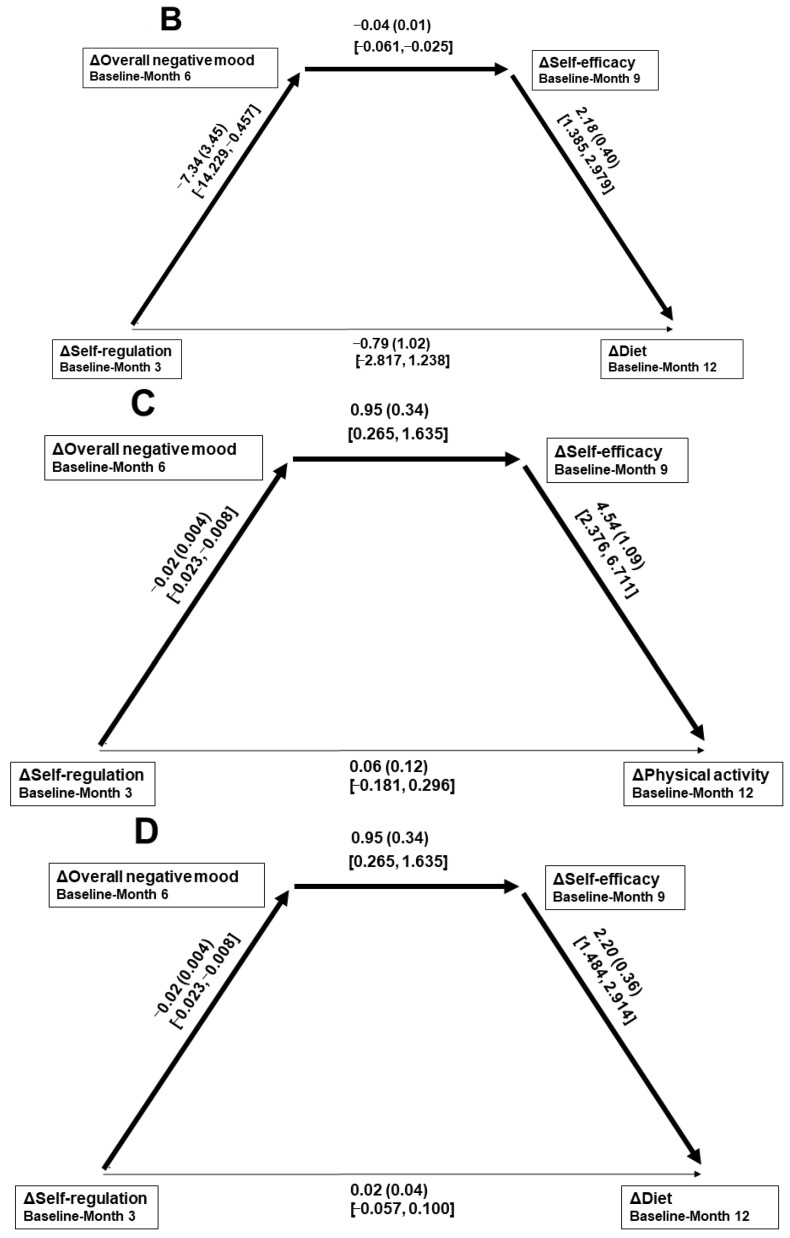
Paths of temporally sequenced psychological changes toward changes in physical activity (**A**,**C**) and dietary behaviors (**B**,**D**) *N* = 73. Δ, change during the designated temporal period. Path data are given as unadjusted beta (its associated standard error) and [95% confidence interval]. A bolded arrow indicates a significant bivariate relationship within the model.

Of the significant paths found in the parent study [42], only those displayed in Figure 1C (B = −0.07, *SE*_B_ = 0.03, 95% CI [−0.140, −0.006]) and Figure 1D (B = −0.03, *SE*_B_ = 0.02, 95% CI [−0.065, −0.005]) were significant within the present sample. Given non-significant relations after accounting for the path variables, both models demonstrated complete mediation as the direct effect of ΔOverallNegMood on ΔPA was B = 0.06, *SE*_B_ = 0.12, 95% CI [−0.184, 0.296], and the direct effect of ΔOverallNegMood on ΔDiet-overall was B = 0.02, *SE*_B_ = 0.04, 95% CI [−0.057, 0.100].

### 3.3. Moderation of the Mood–Self-Regulation Relations Within Significant Paths

Within the subsequent moderation analyses, regarding the overall path displayed in Figure 1C, the ΔOverallNegMood → ΔSelf-reg-Ag relationship was significantly moderated by baseline emotional eating (B = 0.001, *SE*_B_ = 0.001, 95% CI [0.001, 0.002]; IMM B = 0.006, *SE*_B_ = 0.004, 95% CI [0.0001, 0.016]) and anxiety (B = 0.002, *SE*_B_ = 0.001, 95% CI [0.0002, 0.003]; IMM B = 0.007, *SE*_B_ = 0.007, 95% CI [0.0001, 0.026]), but not by the depression score at baseline. Within the path displayed in Figure 1D, the ΔOverallNegMood → ΔSelf-reg-Ag relationship was also significantly moderated by emotional eating (B = 0.001, *SE*_B_ = 0.001, 95% CI [0.0004, 0.002]; IMM B = 0.003, *SE*_B_ = 0.002, 95% CI [0.0001, 0.006]) and anxiety (B = 0.002, *SE*_B_ = 0.001, 95% CI [0.0002, 0.003]; IMM B = 0.003, *SE*_B_ = 0.003, 95% CI [0.0001, 0.011]), but not by depression.

### 3.4. Correlates of Physical Activity and Weight Changes

There was a significant inverse relationship between 3-month changes in PA and OverallNegMood (B = −0.42, *SE*_B_ = 0.11, β = −0.41, *p* < 0.001), but not between baseline PA and OverallNegMood change. Together, 12-month changes in PA and Diet-overall were significantly associated with the observed weight change (*M* = −5.15 kg, *SD* = 4.72, range = −20.70 to 1.81 kg) over 12 months (*R* = 0.38, *R*^2^ = 0.15, *p* = 0.004). That mean weight loss represented a 5.5% reduction from baseline.

## 4. Discussion

This study fulfilled aims to advance a systematic progression of theory-based field inquiry [30,42]. That applied research program was, and continues to be, focused upon addressing treatment-associated changes in mood, self-regulation, and self-efficacy to attain sustained improvements in the key weight-loss behaviors of PA and controlled eating [30]. The present investigation addressed limitations of its parent study by restricting inclusion to women who, at baseline, had not already completed treatment targets related to fruit/vegetable intake (an available proxy for the overall diet [44]) and PA. While each of those behaviors are, both independently and in-combination, predictive of weight loss [75,76], they might not apply well to individuals with high energy intakes unaffected by their completion. In the case of the present research, the sample size of the parent study was reduced by 31% when the additional inclusion criteria were applied. Because the evaluated paths—which were derived from the previous sample based on their significant prediction of 12-month changes in both PA and the overall diet—indicated that interactions between early changes in Self-reg-Ag and OverallNegMood were key, possible theory- and research-based moderators of that relationship (i.e., emotional eating, anxiety, and depression [71]) were assessed. Those analyses were also intended to advance behavioral obesity-treatment contents.

The identified significant improvements in Self-reg-Ag, Self-eff-Ag, and OverallNegMood were expected. The negation of improvements in Self-reg-Ag and Self-eff-Ag beyond the initial 6 months (i.e., baseline–Month 9) was consistent with reductions in adherence of both weight-loss behaviors and weight loss that typically occur after that point [77,78]. Sustaining gains in psychological correlates of such behavioral changes beyond a time when the body starts to resist weight loss, thus diminishing its reinforcing effects [79], remains a challenge [80]. Such behavioral changes should be better addressed within both theory and related research. It has been suggested that weight loss and weight-loss maintenance be considered separately [81], and that a goal of *sustaining* behavioral changes and lost weight between Months 6 and 9 be enacted as a part of treatment curricula [30]. It was posited that this could temper disappointment (and reduced behavioral adherence) related to the expected plateau in weight [5,12]. Definitive analyses of such, and how to structure behavioral treatment content beyond an initial year (i.e., its follow-up processes), will require increased consideration and testing in the future. The 12-month duration of the present research was limited in that area.

Hypothesis 2 was partially supported. Paths toward 12-month changes in PA and Diet-overall were significant for temporally progressive changes (i.e., sequentially over 3, 6, and 9 months) in OverallNegMood → Self-reg-Ag → Self-eff-Ag, but not where early change in Self-reg-Ag was first entered as a predictor of longer-term change in OverallNegMood. Consistent with social cognitive theory [31] and self-efficacy theory [33], psychosocial changes leading to Self-eff-Ag score changes over the longer term was strongly associated with improvements in both of the assessed weight-loss behaviors. This relationship was also supported within studies evaluating the effects of self-efficacy on behavioral changes and weight loss [82]. Given that increased PA has consistently been related to improvements in mood, especially in individuals who are initially low-active [35], the inclusion of participants with minimal PA was particularly justified here. Indications are that, within such a sample, PA should be supported initially for its mood-enhancement potentials that may then serve to facilitate increased self-regulatory skill usage. Both the mood–behavior model [34] and previous research [30] reinforces purposefully targeting that sequence of psychosocial changes. Thus, the paths identified here as significant serve to reinforce treatment foci on self-regulation, mood, and self-efficacy, and address *when* to focus on each as they advance toward long-term changes in PA and eating behaviors. Further, because of the association of early increases in PA with mood enhancements, it is suggested that PA be addressed prior to focusing on dietary changes. That suggestion is supported by coaction theory [39] and studies indicating a carry-over of early PA changes on improvements in eating behaviors and weight [42,83]. Thus, comprehensive evaluation of interactions of elements of the significant paths, focused individually on PA- and diet-related psychological changes, is warranted for extensions of this research.

Within the significant paths, the moderation of the OverallNegMood → Self-reg-Ag change relationship, by baseline scores of emotional eating and anxiety, added another possible dimension to future behavioral treatments. The emotional eating scores [52] were highly variable and difficult to contrast with women, overall, because of a lack of such normative data. However, the mean anxiety score of the present sample was higher than normative values in women across ages and body compositions [51,84]. Although future direct investigation is required, it is possible that early treatment attention paid to PA-associated anxiety reduction will also benefit emotional eating. That relationship has previously been supported [85]. Within the paths identified here as salient, that process should, in turn, increase the self-regulatory skill usage toward improved self-efficacy, weight-loss behavior changes, and lost weight. Although more cumbersome, it is also possible that identifying and counseling high emotional eating and anxiety will be effective for advancing self-regulation.

Corroborating the final hypotheses, and in agreement with most of the previous related research [35], (a) increased PA was significantly associated with reductions on OverallNegMood and (b) 12-month changes in PA and Diet-overall was significantly related to the amount of weight loss during the same period [30,40,86,87]. Although the weight reduction effects were not commensurate with the large effect sizes in the psychosocial variables, the observed mean weight loss of 5.5% suggested that most participants completed sufficient weight loss to induce significant reductions in their health risks [88]. It also indicated that degree of weight loss is comparable to (not significantly different than) the use of GLP-1 medications in similar sample types in real world settings [21]. However, as suggested earlier, explanatory mechanisms differ across those divergent weight-loss intervention types, making direct comparisons largely unsuitable. Additional research will be required to determine if combinations of medications with behavioral theory-derived behavioral approaches (as here) will induce greater, or more sustained, effects on weight than either alone [89]. This is important because it was reported that only 27% of individuals were identified as adherent to prescribed obesity medications at the 12-month point [20].

Although several limitations of this research were already mentioned, there were others that also warrant indicating. For example, the incorporation of a single author conducting analyses could introduce bias. Inclusion of control and/or contrast groups, especially those with more typical educationally focused processes, will help account for behavioral treatment effects related to social support and participant expectations [90]. Also, samples derived from medical referrals, where medical professionals are assertive in their recommendation of their patients’ behavioral obesity treatment participation, might counter bias associated with volunteerism [91]. Tests of generalizability of findings are needed through replications with men, across ethnic/racial make-ups and socioeconomic strata, age groupings, and additional facilities, and also with participants having medical issues beyond obesity. Further validation of the aggregated approach to psychological measurement used here is needed, as well as a greater theoretical basis for such aggregation. Additionally, there was a strong dependence on the accuracy of self-report scales, with possible confounding associated with self-report biases and their longitudinal use. Although cumbersome, more advanced measures of PA (e.g., accelerometry [92]) and the diet (e.g., doubly labeled water [93]) should be considered for extensions of this research. However, although some loss of validity is possible within field environments, it is still recommended for extensions of this research to enhance real-world applicability. This is especially true for community settings where many individuals might benefit in a cost-effective manner [94].

## 5. Conclusions

In conclusion, the present research utilized strong behavioral theory to advance a program of applied research in behavioral obesity treatment. It extended its parent study through the incorporation of a practically relevant sample of women with obesity and better accounted for personal psychological factors in promoting self-regulatory skill usage. Findings related to the investigation of sequential paths improved the timing of emphases on psychosocial factors related to longer-term improvements in essential weight-loss behaviors. Such relations have considerable implications for clinical application, regardless of format (e.g., via individual and group counseling; through written materials; via electronic and/or interactive artificial intelligence delivery). Research should continue seeking refinements in aspects of behavioral obesity treatment contents and consider that modality’s applicability and relative worth when contrasted with, or added to, medical means.

## Figures and Tables

**Table 1:** Descriptive data and gain scores on the assessed psychological and behavioral variables.

Baseline			Month 3		Month 6		Month 9		Month 12	
			[ΔBaseline–Month 3]		[ΔBaseline–Month 6]		[ΔBaseline–Month 9]		[ΔBaseline–Month 12]	
	***M***	***SD***	***M***	***SD***	***M***	***SD***	***M***	***SD***	***M***	***SD***
Self-efficacy	4.52	1.40	6.06	1.63	6.52	1.67	4.02	1.30		
			[1.54]	[1.79]	[2.00]	[1.81]	[−0.50]	[1.65]		
Self-regulation	1.76	0.43	2.50	0.33	2.59	0.32	0.79	0.51		
			[0.75]	[0.56]	[0.83]	[0.52]	[−0.96]	[0.90]		
Total mood disturbance	23.99	14.46	9.56	15.46	4.87	11.53	7.03	11.51		
			[−14.42]	[14.72]	[−19.11]	[16.17]	[−16.96]	[16.92]		
Physical activity	7.82	7.06							30.78	15.27
									[22.96]	[16.21]
Overall diet	3.61	3.08							10.60	4.53
									[6.99]	[5.14]
Emotional eating	28.03	10.45								
Anxiety	5.29	4.29								
Depression	4.34	3.52								

*N* = 73. Δ, change during the designated temporal period (given within brackets). Within the text, scores for Self-efficacy (aggregated) = Self-eff, Self-regulation (aggregated) = Self-reg, Total mood disturbance = OverallNegMood, Physical activity (METs/week) = PA, and Overall diet = Diet.

## Data Availability

The data presented in this study are available on request from the corresponding author due to privacy and ethical reasons.
